# The relationship between agricultural fires and livestock farming

**DOI:** 10.1016/j.heliyon.2024.e40455

**Published:** 2024-11-15

**Authors:** Burak Öztornacı

**Affiliations:** Cukurova University, Department of Agricultural Economics, Adana, Turkey

**Keywords:** Livestock farming, Stubble burning, Panel data, Turkiye

## Abstract

Although many studies have been conducted in recent years on the environmental damage caused by the livestock sector, there are some gaps in terms of possible positive impacts. In this study, in order to investigate the possible positive environmental impacts of the livestock sector, the relationship between agricultural fires and livestock in Turkey between 2012 and 2021 is analyzed. Within the scope of the study, micro-level data, remote sensing datasets and fixed effects panel data method were used. As a result of the analysis at the district level, it was concluded that the development of the livestock sector and the decline in second crop corn production caused by this phenomenon reduced stubble fires. This result reveals that this maturity should be taken into account in future studies on the environmental impacts of the livestock sector.

## 1 Introduction

In recent years, numerous scientific studies have been published regarding the environmental impacts of livestock farming [[1], [2], [3], [4], [5], [6], [7]]. These studies indicate that one of the foremost environmental damages caused by livestock farming is the harm inflicted on the atmosphere through greenhouse gas emissions [[8], [9], [10], [11], [12], [13]]. However, there may be overlooked benefits beyond the environmental damages, particularly those directed towards the atmosphere, caused by livestock farming.

The advancement of the livestock sector, particularly in developing countries, is inducing a shift in the pattern of crop production. The increasing numbers of livestock are associated with a rise in the cultivation of fodder crops. This phenomenon, notably observed in countries such as China, India, and Turkey, reduces environmental and atmospheric damage through the incineration of residues from wheat and maize products [14].

In Turkey, one of the countries where this process has occurred, the number of large livestock increased by 29 %, and the number of small livestock increased by 60 % between 2012 and 2021 (The number of cattle increased by 29 %, buffalo by 81 %, sheep by 65 % and goats by 48 %, respectively) [15]. Similar increases have been observed in the production of forage crops during the same period. Silage corn production increased by 90 %, fodder oats production by 398 %, clover production by 65 %, fodder wheat production by 68 %, sorghum production by 128 %, fodder barley production by 2794 %, and fodder rye production by 7325 % [16]. The development of the livestock sector has had a stimulating effect on the increase in forage crop production.

In recent years, Turkish farmers have shifted their focus towards cultivating forage crops, abandoning certain previously produced products. Foremost among the crops that farmers have left cultivation are wheat and corn. This action is attributed to the fact that in Turkey, farmers can cultivate wheat and corn within the same soil during a single agricultural season, a method commonly known as double cropping [17]. Over the period spanning from 2012 to 2021, the double cropping area for wheat and corn in Turkey has witnessed a reduction of approximately 15 %. Most of these cultivation areas have been replaced with forage crops [18].

Crop rotation involves strategically altering crops within a specified time frame, within the same year, or over multiple years. Within a year, farmers may implement crop rotation by double cropping, wherein more than one crop is planted in the same field [19,20]. This practice often entails burning the fields after harvesting the primary crop to facilitate the cultivation of a secondary crop [17,21]. However, the incineration of agricultural lands gives rise to various environmental challenges. Agricultural fires contribute to air pollution by releasing toxic gases into the atmosphere [22,23]. In nations such as India, a staggering 97 % of atmospheric pollutants stem from the incineration of agricultural residues, prominently encompassing crop remnants such as rice, wheat, and corn [24]. The resultant exposure to these agricultural fires and associated toxic emissions have been linked to adverse health outcomes, including low birth weights, diminished heights, premature mortality, and impairment of cognitive functions [[25], [26], [27], [28]].

This study aims to shed light on overlooked positive impacts of livestock farming despite the adverse effects identified on the environment and human health in recent years. The relationship between Turkey's rapidly evolving livestock sector and stubble fires from 2012 to 2021 is analyzed using remote sensing, plant production, and livestock datasets.

Because the fight against stubble fires is one of the most important agricultural problems for Turkey and it is thought that the livestock sector, which has been developing in Turkey in recent years, may have positive effects on this issue [14]. The main hypothesis of the study is that with the development of the livestock sector, farmers have abandoned double cropping to grow the crops needed by the livestock sector, which may be associated with a decrease in stubble fires. Consequently, this research contributes valuable insights for Turkey and countries reevaluating their policies related to the livestock sector in light of current research findings.

This research contributes to the scientific literature in various ways. It investigates, for the first time, the relationship between livestock farming and stubble fires. Despite numerous studies focusing on the adverse effects of livestock farming, these works overlook that livestock farming mitigates stubble fires [[1], [2], [3], [4], [5]]. Our study utilises national agricultural and remotely sensed data rather than relying on geographically limited and cross-sectional datasets such as farmer surveys. This approach allows us to consider temporal effects.

The subsequent sections of the paper are structured as follows: The second section provides a brief overview of the research region. The third section details the datasets employed in our analysis. The fourth section discusses methodology, followed by results in the fifth section. The sixth section discusses the study's limitations. Finally, the seventh section concludes by highlighting the implications of our findings.

## 2 Research region

**Fig. 1:**
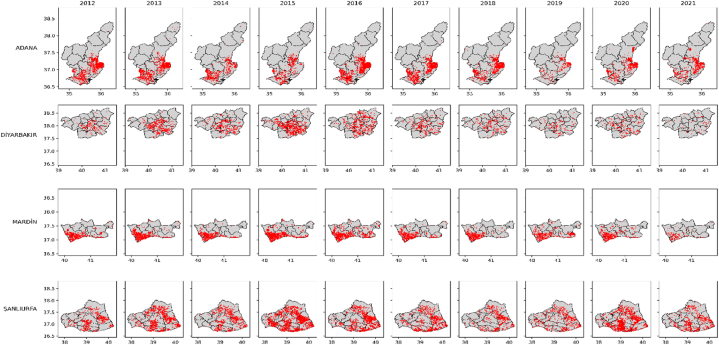
Total number of fires in 4 provinces of Turkey during summer months (June–August).

This study focuses on stubble fires in Turkey, which have been concentrated in four provinces (Adana, Sanlıurfa, Diyarbakır, Mardin) and 48 districts in these four provinces (Fig. 1) [29]. These provinces are also situated within the region where wheat was first domesticated [30,31]. Turkey is endowed with four major rivers in these provinces: the Seyhan, Ceyhan, Euphrates, and Tigris. Over the past 30 years, the region's surface area of irrigated agricultural land has increased sixfold [32].

These provinces are crucial for grain production in Turkey, encompassing 81 % of the wheat and secondary crop maize production areas [18]. The predominant agricultural practices in the region are the primary cause of stubble fires [17,33]. However, in recent years, a decrease has been observed in the cultivation of wheat and secondary crop maize, as well as in stubble fires in this region [18,29]. These crops have been replaced primarily by forage crops. The total area of forage crop cultivation in these four provinces has increased by 110 % between 2012 and 2021 in these four provinces [16]. The primary reason for this change is the growth of the livestock sector in recent years. The total number of large livestock increased by 46 % and small livestock by 70 % between 2012 and 2021 in these four provinces [15].

## 3 Datasets

In this study, we conduct a comprehensive analysis of Turkey, focusing on the period from 2012 to 2021. Our objective is to evaluate the micro-level effects by utilizing district-level variables. Throughout the study period, several central districts underwent subdivision, and to maintain temporal consistency, we aggregate these newly formed districts with their original central districts. In other words, we worked with a total of 480 observation values for 48 districts over a 10-year period.

Primary fire data is sourced from NASA's real-time fire point dataset, FIRMS (VIIRS-NPP 375m) [29]. This dataset classifies fires into three confidence levels: low, nominal, and high. Our analysis focuses on fires with high and nominal confidence levels, excluding low confidence points which likely represent sunlight reflections or minor temperature anomalies [34]. Using the prepared map data, we aggregate the number of fires at the district level, detailing the total and monthly counts per district and year. This aggregation serves as the primary variable for regression analysis. Additionally, to specifically examine stubble fires, we restrict our analysis to fires occurring in June, July, and August.

Corn is a significant factor in the context of stubble burning in Turkey. In the provinces of Adana, Diyarbakır, Mardin, and Şanlıurfa —regions where second-crop corn is predominantly cultivated— planting occurs in June and harvesting takes place in August. This rapid cropping cycle provides farmers with only a few weeks to clear fields for the subsequent planting season, thereby increasing their reliance on stubble burning as a method for field preparation. Therefore, our analysis incorporates areas designated for second-crop corn cultivation to fully capture the impact of these agricultural practices on stubble burning.

We estimate average temperature and total precipitation for June, July, and August using data from the Climate Research Unit (Harris et al., 2020). Additionally, our analysis incorporates data on population changes and livestock numbers within the study area [15,16,18].

## 4 Methodology

This study investigates the intricate interplay between Livestock Farming and Stubble Burning through a comprehensive analysis utilizing panel datasets comprising district-level and year-level observations. The use of this dataset configuration offers considerable advantages for examining the complex relationships between variables, particularly by enabling control for unobserved factors through the application of unit- and time-fixed effects. Given that our dataset consists of panel data, we were able to leverage panel data methodologies, which offer significant benefits. One of the primary advantages of these methods is the ability to account for fixed effects. In this context, two key types of fixed effects were employed: time-fixed effects and units (districts)-level fixed effects. Time-fixed effects account for factors that vary over time but are constant across districts, while district-level fixed effects control for factors that are specific to each district but remain constant over time. By incorporating these fixed effects, we effectively mitigate biases arising from temporal or district-specific influences, thus enhancing the robustness of our analysis. The model's structural framework is delineated as follows:

(1) $$y_{dt}=\delta_{d}+\gamma_{t}+\beta X_{dt}+\varepsilon_{dt}$$

In the context of this study, $y_{dt}$ denotes the aggregate count of fires transpiring within district d during year t. The variable X dt incorporates essential parameters, including temperature, precipitation, livestock populations, human populations, and the area designated for double cropping of wheat and corn. The term $\delta_{d}$ represents the district-fixed effect, pivotal in attenuating latent variables' influence on fire occurrences. Incorporating this control variable is instrumental in mitigating potential biases inherent in assessing independent variables vis-à-vis fire incidents. $\gamma_{t}$ denotes the time-fixed effect, capturing overarching trends and temporally varying shocks that uniformly impact all districts annually. Finally, $\varepsilon_{dt}$ is the error term. The statistics of the variables used in the analysis are shown in Table 1.

**Table 1:** Descriptive statistics.

Variables	Units	Mean	S.D.	N	Specification
Number of fires	Count (3 months)	103.76	8.18	480	*Dependent*
Precipitation	cm (3 months)	25.27	1.18	480	*Independent*
Temperature	^◦^C (3 months)	28.32	0.13	480	*Independent*
Population	Count (thousands)	138.90	11.47	480	*Independent*
Number of livestock	Count (thousands)	128.50	7.27	480	*Independent*
Secondary crop of corn land	da	3028.26	323.14	480	*Independent*

Note: S.D. represents standard deviation and N denotes the total observations.

## 5 Results

**Fig. 2:**
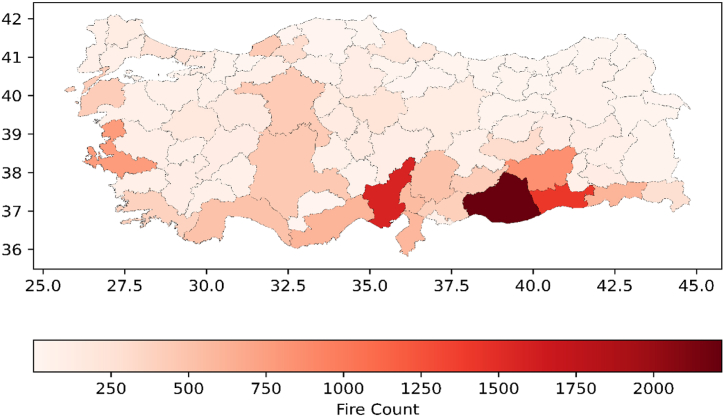
Distribution of fires in Turkiye. Note: Average of ten years (2012–2021).

**Fig. 3:**
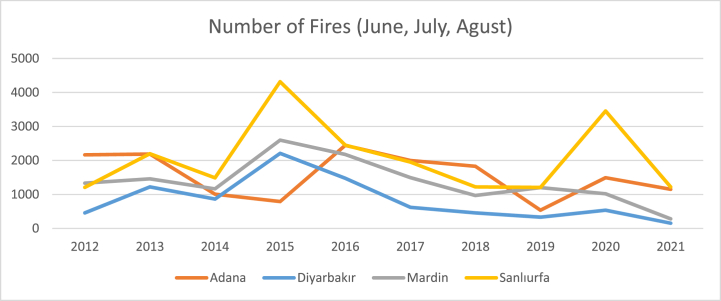
Total number of fires across the 3 months in these 4 cities.

In this study, the effect of livestock sector on stubble fires was examined. First of all, according to the FIRMS dataset, there are 40000 fires in Turkey on average per year, and about a quarter of these fires occur in only 4 cities (Adana, Diyarbakır, Mardin and Şanlıurfa) (Fig. 2). Approximately three quarters of the fires in these four cities are agricultural fires in the summer months (Fig. 3). The main cause of these fires is the second crop corn, which is intensively grown in the region. However, Fig. 3, there is a decreasing trend in the number of stubble fires in these cities. In the region, the second crop corn is being replaced by fodder crops. The main reason for this change is the increasing livestock production in the region.

Although these data provide some insights into this change in the region, it is not possible to establish a direct relationship between animal husbandry and stubble fires. The results of the regression model we used to reveal this relationship are shown in Table 2. In our regression analysis, we used a fixed effects model with year dummies. We focused on 5 variables in total.

**Table 2:** Regression results.

Variables	Regression coefficients
Precipitation	−0.956
	(-1.003)
Temperature	49.524∗∗
	(-18.866)
Population	−0.864∗∗∗
	(0.175)
Number of livestock	−0.268∗∗∗
	(0.091)
Secondary crop of corn land	0.010∗∗
	(0.004)
Cons	−1149.024∗∗
	(-525.991)
Observation	480

Regression analysis revealed no significant relationship between rainfall and stubble fires (−0.956). This is due to the fact that the summer months are quite dry in the region. However, there is a positive and significant relationship between temperature and stubble fires (49.524∗∗). As the temperature increases, the number of fires increases. The effect of population variable is negative and significant (−0.864∗∗∗). Increasing population in the region drives urbanization, leading to a reduction in agricultural land and consequently a decline in fire occurrences [35]. As expected, a negative and significant relationship was calculated between the number of animals, which is the main variable we focus on in this analysis, and stubble fires (−0.268∗∗∗). The increasing number of livestock in the region in recent years has increased the production of fodder crops, increased fodder crop production has reduced the production of second crop maize, and this phenomenon has reduced stubble fires. Accordingly, a positive and significant relationship was found between second crop corn cultivation area and stubble fires (0.010∗∗).

## 6 Discussion

This research demonstrates that the development of the livestock sector is associated with a reduction in stubble fires. Specifically, changes in agricultural production patterns resulting from livestock development are linked to decreased occurrences of stubble fires. Previous studies have identified second crop maize production as a significant contributor to stubble fires [21,36]. Our analysis indicates that the decline in second crop maize cultivation correlates with a reduction in stubble fire incidents, corroborating findings reported in the existing literature. Notably, prior research on stubble fires in Turkey has shown that increased livestock presence correlates with fewer stubble fire incidents, yielding a coefficient of −0.125 [14]. In our analysis, we observe a coefficient of −0.268, which further supports the hypothesis proposed in this study and reinforces the robustness of our research methodology.

The main reason why farmers burn stubble is economic. Producers who grow two crops in a season need to quickly destroy the remains of the first crop to maximize profit. This situation increases the costs of producers [37]. Therefore, producers resort to stubble burning. However, the development of the livestock sector offers producers both an alternative production pattern and the chance to sell the residues of crops such as wheat instead of destroying them.

The penalization method, which is another phenomenon that can reduce stubble burning, is not included in the analysis. Because the fines for stubble burning in Turkey are both very low and often canceled by the courts [14].

Other methods can be proposed to reduce stubble fires. The construction of biomass power plants that purchase crop residues from producers [38], offering producers various financial resources to deal with crop residues [39] and so on. However, these phenomena are not the focus of this research. This research focuses on the fact that livestock farming can have a positive impact on the environment by reducing stubble burning.

## 7 Conclusion

In this study, the possibility that the livestock sector, which has been claimed to cause a lot of damage to the environment in recent years, may also have some overlooked environmental benefits is discussed. For this purpose, the relationship between livestock production and stubble fires was analyzed in four provinces with the highest number of stubble fires in Turkey between 2012 and 2021. The analysis was conducted in detail covering 48 districts in these four provinces. The fixed effects panel data method is used, taking into account other variables that are likely to affect stubble fires. As a result of the analysis, it was concluded that the increasing animal production in the region and the decreasing second crop corn cultivation decreased stubble fires. These results reveal the possibility that this possible effect should be taken into account in studies on animal production in recent years.

The primary limitation of this study lies in the availability of data. Currently, there is no comprehensive or detailed dataset on stubble fires in Turkey. The development and publication of more robust micro-level datasets in the coming years would enable a more nuanced analysis of the environmental impacts of livestock farming. Additionally, there is a lack of data regarding the geographical locations of animal farms in Turkey, which presents a significant gap. Generating such data could prove crucial for future studies aiming to assess the environmental consequences of animal husbandry more accurately. Future researchers should prioritize the establishment of more detailed and geographically referenced datasets related to both agricultural practices and livestock management. Such data will be invaluable for disentangling the complex interactions between livestock presence and environmental outcomes, including stubble fires. Collaborative efforts with governmental agencies, environmental organizations, and academic institutions can facilitate the development of these datasets. Furthermore, adopting interdisciplinary approaches that combine environmental science, agricultural economics, and geospatial analysis may enhance the depth and relevance of future studies on the environmental impacts of animal production.

## Data availability

All the datasets are publicly available.

## Additional information

No additional information is available for this paper.

## Declaration of competing interest

The authors declare that they have no known competing financial interests or personal relationships that could have appeared to influence the work reported in this paper.
